# Evidence for a role of adaptive immunity in Tau‐mediated neurodegeneration

**DOI:** 10.1002/alz.087778

**Published:** 2025-01-03

**Authors:** David M. Holtzman

**Affiliations:** ^1^ Washington University School of Medicine, Saint Louis, MO USA

## Abstract

**Background:**

Recent evidence suggests both the innate and adaptive immune system are involved in the pathogenesis of Alzheimer’s disease. We recently showed that the number of T cells, especially cytotoxic T cells, are increased in areas of tauopathy both in an animal model of tau‐mediated neurodegeneration as well as in the AD brain. We also found that depletion of T cells as well as microglia were both strongly neuroprotective in P301S tau transgenic mice (PMID: 36890231). We have been further characterizing T cells in this model as well assessing for other evidence for a role of the adaptive immune system in this model.

**Method:**

We crossed P301S mice with Rag2KO mice that lack both T and B cells and then analyzed the mice at 9.5 months of age.

**Result:**

P301S/Rag2KO mice have few to no T cells in the brain but also have strong suppression of the brain’s innate immune response and have significantly less brain atrophy and loss of synapses and compared to P301S mice.

**Conclusion:**

This provides further evidence of a role for the adaptive immune response in tau‐mediated neurodegeneration.

